# Supplementary material for: Arab2Vec: An Arabic word embedding model for use in Twitter NLP applications
Source: PLoS One. 2025 Aug 29;20(8):e0328369. doi: 10.1371/journal.pone.0328369 (PMC12396693; doi:10.1371/journal.pone.0328369)
Supplement: Attachment — Submitted filename: Review.docx [file pone.0328369.s001.docx]

# Review, 20 Feb. 2023

Manuscript Number: PONE-D-23-01033

Manuscript Title: Arab2Vec: An Arabic word embedding model for use in Twitter NLP applications

## Summary

In this work, the authors present their work on learning distributed vector representation (also called word embeddings) on Tweets written in Arabic using the Word2Vec algorithm by Mikolov et al. Their work made several improvements compared to existing, similar word embeddings. Their word embeddings were trained on larger quantities and more recent Tweets compared to previous work, they learned embeddings on emojis, which none of the previous embeddings for Arabic tweets did, and used a variation of Word2Vec that improved training time and accuracy in the evaluation step.

Their embeddings were evaluated in comparison to existing embeddings in a qualitative and quantitative fashion. The evaluation consisted of: (i) Comparing the number of overall recognized words as a measure of completeness, (ii), qualitative assessments of word similarity in the embedding space coming from manually selected words, (iii) a visual assessment of clusters of word embeddings for negative and positive sentiment words, (iv) a visual assessment of the cluster quality of words belonging to the same named entity, (v) and finally testing the performance of several classification algorithms using several word embeddings to identify which embedding performs the best showing that their proposed embeddings yielded the best results in most tasks.

## Overall feedback

+ The manuscript tells a clear and consistent story that is easy to follow

+ The authors presented a diverse and compelling evaluation strategy

+ They motivated and described their work in a succinct manner

+ I believe it is important that the authors worked on Arabic word embeddings since Arabic does not enjoy as much attention as other languages in the research community despite being spoken by relatively many people

- The paper has strong grammatical deficits

- The section on Literature Survey is wordy and unstructured

- The models and code are not accessible with the provided GitHub link

I believe that the paper, as I understood it, meets the criteria to be published and thus, I would suggest revising it, albeit several points would need to be addressed. The paper is technically sound and meets the scientific standard.

The grammatical deficits stand out negatively and diminish my confidence in the author’s work as I cannot fully rely on the content which may be ambiguous due to grammatical errors. I strongly recommend the authors to seek help either from peers or professionals to improve the language of the paper such that reviewer and readers in the case of acceptance can focus on the content of your work.

In the following, I will chronologically mention all points that require amendment in my opinion but not mentioning every grammatical error as I am not an editor:

### Cover Page

- According to the official website, Indian Institute of Technology Madras is written without a hyphen before Madras
- The word Limerick is duplicated in the name of the University of Limerick

### Abstract

- You write “**Any** analysis of Twitter data sets requires a word embedding model to transform words into numbers that can be processed using machine learning (ML) algorithms”. This is not correct. Other vectorization methods like bag-of-words can also be used. Please re-write.
- “[…] be used in Twitter natural language processing (NLP) applications.” Do you mean “Twitter-based”?
- “[…] superior performance in terms of number of recognized words and accuracy”. It is not clear to what accuracy refers. Make clear what you refer to, i.e., classification tasks on known data sets. Also, you did not measure accuracy but F1. Consider rephrasing.
- “In all, we produce nine different versions of Arab2Vec […]”. Please write in past tense as this already done. This also applies to the rest of the manuscript.
- “[…] and Experiments to validate […]”. Experiments should be written be written in lowercase.

### Introduction

- Line 2: You reuse “huge” shortly after it has been used in the abstract. Please change for stylistic reasons.
- Line 5: In American English, the first word after a colon is capitalized if it begins a complete sentence <https://www.grammarly.com/blog/capitalization-after-colons/>.
- Line 8: Can you give an example of sentences that are different due to dialectics?
- Line 9: As mentioned above, you can use other techniques to vectorize text data besides word embeddings.
- Line 12: Help ML models with what?
- Line 26: You write about recognizing English words. How did you evaluate their recognition, which advantage brings it to recognize English words? Please elaborate why this is a feature and not an artifact.
- Line 27: You tested your data on two data sets. However, my research showed that there are more data sets on which you can evaluate. Can you elaborate why you did not use them? Find some in section 5.2 from <https://aclanthology.org/C16-1228.pdf>, in section 4.1 of <https://arxiv.org/pdf/2003.00104.pdf>
- Lines 40-46: Why do you write section in uppercase and why do your write “forth” in lowercase in line 42? Why are you missing omitting using an article for Section in this paragraph?

### Literature Survey

This section is missing structure. I understood that you cited related research on Arabic word embeddings, highlighted the performance of these embeddings in several NLP-related tasks, and mentioned pre-processing strategies. However, motivation and structure of this section are unclear. For example, in lines 78-89, you wrote about NLP applications using embeddings and what kind of machine learning models were used to for these tasks, and how they performed. It is not clear why you are mentioning this, as I am expecting a paper on embeddings and not on sentiment analysis. Only later in the paper, when I read the section on evaluation, I understand that you were giving an overview on model performance with existing embeddings and evaluation strategy for this paper. Please make this clearer.

I suggest starting with a rough overview of the section by outlining the intention of the literature survey, namely listing similar work on word embeddings, existing evaluation and pre-processing strategies, and the current performance of these models as the reference for the author’s proposed embeddings.

Finally, I am missing some mentioning of relevant other work. For example, <https://aclanthology.org/L18-1577.pdf> built a word embedding using even more tweets. I think it would be interesting to compare the performance to this embedding. Also, I am missing the comparison to transformer-based approaches to embed words. <https://arxiv.org/pdf/2003.00104.pdf> and others have open-sourced their work on this. It would be particularly interesting to find out how your word2vec approach compares to the currently more popular transformer-based approach. This would help answer where word2vec approaches may be more senseful than transformer-based approaches. For your interest, I found this post, where they extract embeddings from pre-trained transformers: <https://mccormickml.com/2019/05/14/BERT-word-embeddings-tutorial/#1-loading-pre-trained-bert>

### Data pre-processing

- Line 141: How did you deduplicated? Did you check if strings were exactly the same or did you use some kind of fuzzy matching?
- Line 150-154: Why did you not use special tokens like <LINK> instead of the Arabic word for link which may be used in vastly different contexts? The same question applies to the Twitter mentions for, e.g., persons or brands.
- Did you not perform any other forms of pre-processing like normalization?
- How did you tokenize your data?

### Effort in these steps

- Line 181: Do you mean the window function? Does this mean, you examine 300 words before and after the word of interest? To me, word count threshold sounds like a trimming rule to remove words that occur less than 300 times. Why is this threshold different for both models? Please be more accuracte. How large are the embeddings, how large was the context window? Ideally, list all hyperparameters and put them into the appendix.
- Line 184: You treat the whole corpus as tri-grams? Isn’t it more practical to only consider tri-grams when words frequently appear in bi- or tri-grams like here: <https://radimrehurek.com/gensim/models/phrases.html#module-gensim.models.phrases>

### Model evaluation and comparison

- Line 194: According to the AraVec paper, there is the SemEval-2017 Semantic Textual Similarity Task. Why are you not using this benchmark but instead used your own words with no clear expectation on the output?
- Line 203: How did you run this test? It reads as if you ran your model against a test set of words. Did you do this or did you simply count the number of keys in your key-to-embedding-dictionary?
- Table 1: Rename to column to “Number of recognized words”. Also, please use book tabs style tables. Vertical lines are not recommended <https://nhigham.com/2019/11/19/better-latex-tables-with-booktabs/>. The same applies to the other tables.
- Fig. 2: Personally, I like the Arabic font from this plot better than in the manuscript as I think it is more readable and more modern. However, other papers also use the same font for Arabic in the main text as you so you can proceed as you like. Please do not use black background for your figures. The readability is poor and it is not recommended (<https://www.ncbi.nlm.nih.gov/pmc/articles/PMC6151470/>). The same applies to the other figures.
- Line 231: Did the other models fail to recognize emojis because they were removed during preprocessing? Please explain what “fail” in this case means.
- Line 234-: What clustering method did you use? Why?
- Table 2: This table is not readable. You don’t provide any description on what are the positive and what are the negative words. Also, it may be a good idea to translate the words for the non-Arabic speaking audience. You did it in the figures on word similarity, why not everywhere? Second, there are repeated words. رائع and مهاري are duplicated.
- Line 244: If the words in the word sentiment clustering task are not available for ArWordVec, why are you not choosing a different set of words for the task? In the AraVec paper, they used a sentiment lexicon. Couldn’t you also use one to select words that all models recognize?
- Table 3: Same formatting should be applied as mentioned above and please add column names. The table caption just says “Caption” and is below and not on top of the table. Please revise, this is a gross error.
- Fig. 8: I suggest you draw circles around the clusters and annotate the circle to show which entities are captures in the cluster. Ideally, you can highlight words that are wrongly clustered to allow readers quickly assess the quality of the clusters. This plot is very hard to read because of the black background, because words overlap, and non-Arabic speaking users cannot assess the quality of the cluster. Please consider the suggested modifications.
- Table 4: Please move to appendix
- Table 5: Why are you not using the same highlighting procedure as in Table 6 by using italics to show the best performing model? Although, I personally prefer other formatting like bold.
- Table 8: Please round all values to two digits.
- Line 286: Do you have an explanation why ML models performed better with ArWordVec CBOW? This contradicts the premise of the paper that more Tweets and Skip gram + negative sampling is producing the best results.
- Table 8: Why are you reporting results on a validation data set? Shouldn’t it be on a test data set like in Table 9?
- Table 9: The table caption say accuracy but the columns say F1. What is it?
- Line 275-: What kind of test, validation, train split did you use? Please explain your training procedure, model parameters, and other details and put them into the appendix for maximal reproducibility
- Line 288: It should say, “were” not used. The embeddings were already created and they did not contain the skip-gram NS strategy, thus you could not show the performance, right?
- Line 299: Was it specifically trained to work with emojis or did you simply not remove them during pre-processing like the other models did. I asked this early and I would like to have clarification how other models treated emojis compared to you.
- Line 304: You cited work that showed that Glove did not perform very well compared to Word2Vec and fastText. Why would you still try Glove? Why are you not mentioning transformer-based or other bi-directional embeddings as potential future approaches?
