# Supplementary material for: Arab2Vec: An Arabic word embedding model for use in Twitter NLP applications
Source: PLoS One. 2025 Aug 29;20(8):e0328369. doi: 10.1371/journal.pone.0328369 (PMC12396693; doi:10.1371/journal.pone.0328369)
Supplement: Attachment — Submitted filename: Review Response.docx [file pone.0328369.s002.docx]

Dear Editor-in-chief (PLOS ONE):

I am pleased to submit our revised article titled “Arab2Vec: An Arabic word embedding model for use in Twitter NLP applications’’ by Abdelrahman Hamdy, Ayman Youssef and Conor Ryan] for your consideration.

We have addressed all reviewer comments as noted below.

# Reviewer 1

1. (Abstract) Can you incorporate why Arabic Twitter data sets are particularly interesting to be highly researched?

The reason that Arabic Twitter data sets are important is the large number of Arabic users on Twitter, and it is estimated that around 186 million Arabic users add to Twitter Arabic tweets.

This reason is mentioned in the introduction now it is added to the abstract.

2. The data collection and data pre-processing steps are well described, but how the Arab2Vec models were trained is very superficial. Which optimization algorithm and configurations did you use? How did you split training/validation sets for Tables 6 and 8? It would be helpful to include in the GitHub repository the codes for scrapping and training of the models as well, not only the trained models. Please make clearer the difference between validation and test sets. For a non-experienced reader this might be unclear.

The GitHub repository contains all the codes for scraping and training the models. We didn’t hide any piece of code. However, the repository was private in the first review round. In this round, we changed its state to public.

3. You mention that you trained nine Arab2Vec models, but Tables 5, 6, 8 and 9 only show three of
them. Please specify exactly which are being shown. Is there a reason why you don’t provide the
results for the other six models? I suppose this wouldn’t require too much extra effort.

There is no extra effort in running the other six models. However, to our understanding, the results of these three models are enough to prove the effectiveness of the proposed model.
4. Please include an English translated version of Table 2, Figs 6, 7, 8 and 9.

We added an English translation for Table 2; however, in our opinion, adding an Arabic translation to Figures 6,7,8 and 9 will make them messy. So, we made the following modifications:

1- Change the background colour to grey.
2- Added circles over each cluster to differentiate the words coming from each cluster to help non-Arabic speakers understand the figures.

We pointed out words that overlap between different clusters
5. How did you choose 25 for the hidden dimension of the LSTM? Have you tried other values?

These values were chosen depending on similar work from literature.
6. Section “Effort in these steps” could be renamed to “Computational effort in these steps”. You
also need to give the specifications of the system (GPU, processor, RAM, etc…).

Fixed the title of the section was changed. System information
7. Line 288 which is in bold. This information is very important and should be made clear much earlier in the paper.
8. Please revise the English:
a. Line 184: “of 100. where tri-gram”

Fixed where removed
b. Line 250: “vehicle types The”

Fixed
c. Figs 8 and 9 captions: “named”

Fixed
d. Table 5: “GB F!”

This is the abbreviation for gradient boost F1 score. Fixed
e. Line 280: “One again”

Fixed and changed to once again
f. Line 302: "models we compare with Arab2Vec is being released"

Fixed

Another contribution of this work is that we provide 288 models that were trained with negative sampling that wasn’t introduced in the previous 289 models from the literature (AraVec and AraWordVec). Arab2Vec is being released to the 290 NLP and AI communities for research purposes.

Reviewer 2

# Review, 20 Feb. 2023

Manuscript Number: PONE-D-23-01033

Manuscript Title: Arab2Vec: An Arabic word embedding model for use in Twitter NLP applications

## Summary

In this work, the authors present their work on learning distributed vector representation (also called word embeddings) on Tweets written in Arabic using the Word2Vec algorithm by Mikolov et al. Their work made several improvements compared to existing, similar word embeddings. Their word embeddings were trained on larger quantities and more recent Tweets compared to previous work, they learned embeddings on emojis, which none of the previous embeddings for Arabic tweets did, and used a variation of Word2Vec that improved training time and accuracy in the evaluation step.

Their embeddings were evaluated in comparison to existing embeddings in a qualitative and quantitative fashion. The evaluation consisted of: (i) Comparing the number of overall recognized words as a measure of completeness, (ii), qualitative assessments of word similarity in the embedding space coming from manually selected words, (iii) a visual assessment of clusters of word embeddings for negative and positive sentiment words, (iv) a visual assessment of the cluster quality of words belonging to the same named entity, (v) and finally testing the performance of several classification algorithms using several word embeddings to identify which embedding performs the best showing that their proposed embeddings yielded the best results in most tasks.

## Overall feedback

+ The manuscript tells a clear and consistent story that is easy to follow

+ The authors presented a diverse and compelling evaluation strategy

+ They motivated and described their work in a succinct manner

+ I believe it is important that the authors worked on Arabic word embeddings since Arabic does not enjoy as much attention as other languages in the research community despite being spoken by relatively many people

- The paper has strong grammatical deficits

The grammar of the paper has been thoroughly revised by a native English speaker

- The section on Literature Survey is wordy and unstructured

- The models and code are not accessible with the provided GitHub link

There was a mistake that the Github link is still private. We currently changed it to public to allow reviewers to see the codes and models of our work.

I believe that the paper, as I understood it, meets the criteria to be published and thus, I would suggest revising it, albeit several points would need to be addressed. The paper is technically sound and meets the scientific standard.

The grammatical deficits stand out negatively and diminish my confidence in the author’s work as I cannot fully rely on the content which may be ambiguous due to grammatical errors. I strongly recommend the authors to seek help either from peers or professionals to improve the language of the paper such that reviewer and readers in the case of acceptance can focus on the content of your work.

In the following, I will chronologically mention all points that require amendment in my opinion but not mentioning every grammatical error as I am not an editor:

### Cover Page

- According to the official website, Indian Institute of Technology Madras is written without a hyphen before Madras

Hyphen removed

- The word Limerick is duplicated in the name of the University of Limerick

A comma is added as it means University of Limerick in Limerick city (a comma was missing)

### Abstract

- You write “**Any** analysis of Twitter data sets requires a word embedding model to transform words into numbers that can be processed using machine learning (ML) algorithms”. This is not correct. Other vectorization methods like bag-of-words can also be used. Please re-write.

The sentence was rewritten to the following statement:

Word embedding models are a key component in the analysis of Twitter data sets. As they are considered one of the important methods of transforming words into numbers that can be processed using machine learning (ML) algorithms

- “[…] be used in Twitter natural language processing (NLP) applications.” Do you mean “Twitter-based”?

The sentence was rewritten, and the word based was added

- “[…] superior performance in terms of number of recognized words and accuracy”. It is not clear to what accuracy refers. Make clear what you refer to, i.e., classification tasks on known data sets. Also, you did not measure accuracy but F1. Consider rephrasing.

The sentence was rewritten

the model is compared with existing models from the literature, and results demonstrate superior performance in terms of the number of recognized words and F1 score for classification tasks with known data sets, as well as the ability to work with emojis.

- “In all, we produce nine different versions of Arab2Vec […]”. Please write in past tense as this already done. This also applies to the rest of the manuscript.

Done

- “[…] and Experiments to validate […]”. Experiments should be written be written in lowercase.

fixed

### Introduction

- Line 2: You reuse “huge” shortly after it has been used in the abstract. Please change for stylistic reasons.

Done

- Line 5: In American English, the first word after a colon is capitalized if it begins a complete sentence <https://www.grammarly.com/blog/capitalization-after-colons/>.

Fixed

- Line 8: Can you give an example of sentences that are different due to dialectics?

There are many words in Arabic that have different meanings depending on dialectics; for example, الجَدّ in Arabic means grandfather, while الجِدّ means diligence.

- Line 9: As mentioned above, you can use other techniques to vectorize text data besides word embeddings.

The sentence was rewritten

- Line 12: Help ML models with what?

The sentence is changed to needed by ML models.

- Line 26: You write about recognizing English words. How did you evaluate their recognition, which advantage brings it to recognize English words? Please elaborate why this is a feature and not an artefact.

Sometimes the tweets contain English symbols because the Arabic tweet writers may use English words or symbols inside the tweets, such as A+.

- Line 27: You tested your data on two data sets. However, my research showed that there are more data sets on which you can evaluate. Can you elaborate why you did not use them? Find some in section 5.2 from <https://aclanthology.org/C16-1228.pdf>, in section 4.1 of <https://arxiv.org/pdf/2003.00104.pdf>

We agree with the reviewer that there are many data sets and problems to test our developed model against. However, in this paper, we are just introducing the paper to the public and testing it with two different data sets just to prove the performance of the model. The authors provide the models for others to test in other different applications to evaluate them.

- Lines 40-46: Why do you write section in uppercase and why do your write “forth” in lowercase

All section words are changed to lowercase

- in line 42? Why are you missing omitting using an article for Section in this paragraph?

The paragraph has been revised

Literature Survey

This section is missing structure. I understood that you cited related research on Arabic word embeddings, highlighted the performance of these embeddings in several NLP-related tasks, and mentioned pre-processing strategies. However, motivation and structure of this section are unclear. For example, in lines 78-89, you wrote about NLP applications using embeddings and what kind of machine learning models were used to for these tasks, and how they performed. It is not clear why you are mentioning this, as I am expecting a paper on embeddings and not on sentiment analysis. Only later in the paper, when I read the section on evaluation, I understand that you were giving an overview on model performance with existing embeddings and evaluation strategy for this paper. Please make this clearer.

I suggest starting with a rough overview of the section by outlining the intention of the literature survey, namely listing similar work on word embeddings, existing evaluation and pre-processing strategies, and the current performance of these models as the reference for the author’s proposed embeddings.

A starting section for the literature survey is added to it

Finally, I am missing some mentioning of relevant other work. For example, <https://aclanthology.org/L18-1577.pdf> built a word embedding using even more tweets. I think it would be interesting to compare the performance to this embedding.

The proposed work by reviewer is not word embedding model! That the proposed model can be compared with

Also, I am missing the comparison to transformer-based approaches to embed words. <https://arxiv.org/pdf/2003.00104.pdf> and others have open-sourced their work on this. It would be particularly interesting to find out how your word2vec approach compares to the currently more popular transformer-based approach. This would help answer where word2vec approaches may be more senseful than transformer-based approaches. For your interest, I found this post, where they extract embeddings from pre-trained transformers: <https://mccormickml.com/2019/05/14/BERT-word-embeddings-tutorial/#1-loading-pre-trained-bert>

In this work, we propose a new model for word embeddings of Arabic twitter words. That is the focus of this research is to propose nine different models with different sizes to work in this research area. In the context of this research, a comparison is made to show the superior performance of the proposed nine models with respect to similar models in the literature. We want to thank the reviewer for his valuable comment, but we feel that comparing our work with pre-trained transformers could be a research extension problem for this work that we can study in future as it will require running more experiments and analysis to reach satisfied results.

### Data pre-processing

- Line 141: How did you deduplicated? Did you check if strings were exactly the same or did you use some kind of fuzzy matching?

We mean here that the same tweets were written to a database we use for training the model. Thus, all we need is a string matching to remove such tweets

- Line 150-154: Why did you not use special tokens like <LINK> instead of the Arabic word for link which may be used in vastly different contexts? The same question applies to the Twitter mentions for, e.g., persons or brands.

In these steps, we are following the same steps of work done in literature (see for example, the AraVec paper); you will find they are using this approach. Also, the word <رابطويب> is rarely used in tweets. Also, the word <حسابشخصي>. Actually, both words are a combination of two Arabic words, which makes them a special token, as the reviewer suggests

- Did you not perform any other forms of pre-processing like normalization?

All steps of pre-processing were described in the paper. We don’t perform normalization to words as we wanted the model to be trained on different words as they are and not trained only on the root of words.

- How did you tokenize your data?

With the tree bank word tokenizer library in Python.

### Effort in these steps

- Line 181: Do you mean the window function? Does this mean, you examine 300 words before and after the word of interest? To me, word count threshold sounds like a trimming rule to remove words that occur less than 300 times. Why is this threshold different for both models? Please be more accurate. How large are the embeddings, how large was the context window? Ideally, list all hyperparameters and put them into the appendix.

The Word2Vec model includes a parameter called the "word count threshold," which helps to eliminate infrequent words from the training data. This is important to reduce noise and improve the quality of the resulting word vectors. The threshold can be set to a minimum number of occurrences, below which words are discarded from the training data. In our analysis, we used different word count thresholds of 100 and 300 to generate models of different sizes that may be suitable for different applications or users. We also found that even rare or infrequent words that occurred at least

- Line 184: You treat the whole corpus as tri-grams? Isn’t it more practical to only consider tri-grams when words frequently appear in bi- or tri-grams like here: <https://radimrehurek.com/gensim/models/phrases.html#module-gensim.models.phrases>

In addition, we aimed to reduce human involvement and allow the model more freedom by not limiting it based on smaller n-grams. This was important because Arabic words have multiple trigrams that do not correspond to bigrams, such as the names of the prophet Muhammad's companions, as well as other Arabic names and hashtags. Limiting the model to smaller n-grams could potentially affect our results. However, we also created a smaller model based only on unigrams to cater to different users and applications

### Model evaluation and comparison

- Line 194: According to the AraVec paper, there is the SemEval-2017 Semantic Textual Similarity Task. Why are you not using this benchmark but instead used your own words with no clear expectation on the output?

For testing the models, there are a number of tasks used in different literature. The authors can add a test for every data set mentioned in the literature. The application we are using to test the models is a data set generated in one previous related work of the authors. So, it is interesting to test our models on the same data set. As such, we don’t have clear expectations on the output. We are testing different models we compare with the literature to compare.

- Line 203: How did you run this test? It reads as if you ran your model against a test set of words. Did you do this or did you simply count the number of keys in your key-to-embedding-dictionary?

No, we didn’t run any tests. We just used a Python instruction to find this number directly

(put instruction or explanation here )

- Table 1: Rename to column to “Number of recognized words”. Also, please use book tabs style tables. Vertical lines are not recommended <https://nhigham.com/2019/11/19/better-latex-tables-with-booktabs/>. The same applies to the other tables.

Fixed

- Fig. 2: Personally, I like the Arabic font from this plot better than in the manuscript as I think it is more readable and more modern. However, other papers also use the same font for Arabic in the main text as you so you can proceed as you like. Please do not use black background for your figures. The readability is poor and it is not recommended (<https://www.ncbi.nlm.nih.gov/pmc/articles/PMC6151470/>). The same applies to the other figures.

Each of Figure 6 – 9 has been given a different background to make them more readable.

Line 231: Did the other models fail to recognize emojis because they were removed during pre-processing? Please explain what “fail” in this case means.

That is correct. The previous models in the literature were not trained to recognize emojis. This is because the authors neglected the importance of Emojis in the Arabic context. However, in our opinion, emojis should be considered an important feature of any embedding model. In our proposed work, we propose not to remove emojis or replace them (negative emoji, positive emoji).

- Line 234-: What clustering method did you use? Why?

K means clustering algorithm similar to literature. Also, there is no need for a more complex clustering algorithm

- Table 2: This table is not readable. You don’t provide any description on what are the positive and what are the negative words. Also, it may be a good idea to translate the words for the non-Arabic speaking audience. You did it in the figures on word similarity, why not everywhere? Second, there are repeated words. رائع and مهاري are duplicated.

Fixed a new table was added with corrections and translation

- Line 244: If the words in the word sentiment clustering task are not available for ArWordVec, why are you not choosing a different set of words for the task?

We wanted to prove a deficiency in the ArWordVec model proposed in the literature that it was trained on a small number of words compared to our model.

- In the AraVec paper, they used a sentiment lexicon. Couldn’t you also use one to select words that all models recognize?

The AraWordVec was trained using a low number of words; this rather limits our options in selecting a set of words that belong to different clusters to test the three models on them. In our opinion, our model is clearly superior to AraWordVec, so trying to select some words that AraWordVec can cluster is unnecessary. The available words set for clustering are recognized by both Arab2Vec and AraVec, which enables us to compare our model against the famous Aravec model from literature.

Table 3: Same formatting should be applied as mentioned above and please add column names. The table caption just says “Caption” and is below and not on top of the table. Please revise, this is a gross error.

Fixed according to reviewer comments.

- Fig. 8: I suggest you draw circles around the clusters and annotate the circle to show which entities are captures in the cluster. Ideally, you can highlight words that are wrongly clustered to allow readers quickly assess the quality of the clusters. This plot is very hard to read because of the black background, because words overlap, and non-Arabic speaking users cannot assess the quality of the cluster. Please consider the suggested modifications.

The authors thank the reviewer for this comment. We agree, the figures needed more clarification. So, we made the following modifications:

3- Change the background colour to grey.
4- Added circles over each cluster to differentiate the words coming from each cluster to help non-Arabic speakers understand the figures.
5- We pointed out words that overlap between different clusters.

- Table 4: Please move to appendix

We want to thank the reviewer for his valuable comment. In our opinion, the hyperparameters of the model are usually presented in the body of the paper, and there is no need for an appendix

- Table 5: Why are you not using the same highlighting procedure as in Table 6 by using italics to show the best performing model? Although, I personally prefer other formatting like bold.

We added the same highlighting procedure produced in Table 6 to other tables as well.

- Table 8: Please round all values to two digits.

Done according to the reviewer's comments

- Line 286: Do you have an explanation why ML models performed better with ArWordVec CBOW? This contradicts the premise of the paper that more Tweets and Skip gram + negative sampling is producing the best results.

That is the result we got from running experiments that in a single case in all experiments ArWordVec has superior performance with the ML model only. This doesn’t mean that it has superior performance over the proposed model. Just for certain applications, the model can give a small error because it was trained on a small number of words, and it happens that this application doesn’t need a variety of words from the embedding model.

The proposed models are provided in public respiratory for researchers to further test in different applications compared with models from the literature.

- Table 8: Why are you reporting results on a validation data set? Shouldn’t it be on a test data set like in Table 9?

This just a typo in writing. Fixed.

- Table 9: The table caption say accuracy but the columns say F1. What is it?

The F1 table caption is fixed.

- Line 275-: What kind of test, validation, train split did you use? Please explain your training procedure, model parameters, and other details and put them into the appendix for maximal reproducibility

All codes we wrote for running experiments are available in Github Respiratory.

- Line 288: It should say, “were” not used. The embeddings were already created and they did not contain the skip-gram NS strategy, thus you could not show the performance, right?

Yes, the previous work introduced in the literature didn’t introduce skip-gram NS strategy models, so we couldn’t compare our trained models with them. And this is considered a contribution to our work.

- Line 299: Was it specifically trained to work with emojis or did you simply not remove them during pre-processing like the other models did. I asked this early and I would like to have clarification how other models treated emojis compared to you.

The main idea of our work is end to end model. We don’t make much pre-processing to the input data. So, in our work, we didn’t remove emojis and instead relied on the large data used to train the model, helping us develop a model that can work with text along with emojis.

- Line 304: You cited work that showed that Glove did not perform very well compared to Word2Vec and fastText. Why would you still try Glove? Why are you not mentioning transformer-based or other bi-directional embeddings as potential future approaches?

We agree with the reviewer that a comparison of our model Arab2Vec with other approaches, like transformer-based, could be a direction for future work. The future work sentence was updated accordingly.
