# Supplementary material for: Arab2Vec: An Arabic word embedding model for use in Twitter NLP applications
Source: PLoS One. 2025 Aug 29;20(8):e0328369. doi: 10.1371/journal.pone.0328369 (PMC12396693; doi:10.1371/journal.pone.0328369)
Supplement: Attachment — Submitted filename: Review_declet_2.docx [file pone.0328369.s003.docx]

2nd Review, 16 December 2023

Manuscript Number: PONE-D-23-01033R2

Manuscript Title: Arab2Vec: An Arabic word embedding model for use in Twitter in NLP applications

Summary

This is the second review of the manuscript. The authors did not redo or add experiments for this revised version. Thus, the manuscript’s overall structure is unchanged. For completeness, I will reuse parts of my summary of the author’s contribution from my first review.

The authors present their work on learning distributed vector representation (also called word embeddings) on Tweets written in Arabic using the Word2Vec algorithm by Mikolov et al. Their word embeddings were trained on larger quantities and more recent Tweets compared to previous work. Furthermore, the authors used a variation of Word2Vec that improved the embedding’s accuracy that comparable work did not use. Their embeddings were evaluated in comparison to two existing embeddings in a qualitative and quantitative fashion showing that their embeddings performed better than two comparable embeddings.

How to proceed

I suggest a second round of revision. The author’s work is relevant, novel, and their experimental approach is up to standards and therefore should be published. Their updated literature review is now more readable. They also addressed most points adequately. Still, the text is at times jarring to read due to grammatical errors. The tables and figures still look sluggish despite the reference-backed feedback I gave. Lastly, the authors addressed a portion of my remarks only in the Author's Response To Reviewer Comments. For the next revision, I urge the authors to address each point in their manuscript.

Feedback

You can find my recommendations for this manuscript in the following section. You will find a summary of the bigger and smaller issued followed by specific points ordered chronologically.

Major Issues

- Failed to address points from the last review in the manuscript itself.

Minor Issues

- Poor design of tables and figures.
- Several grammatical errors.

Introduction

- Lines 5 and 6: Grammatical errors.
- Line 6: I asked in the first round of revision to give examples of dialectics in Arabic. Please explain this also in the manuscript not only in your response letter. Also, dialectic may be the wrong word. Do you mean dialects? Dialectic is a way of philosophical reasoning by assuming contrary positions.
- Line 11: Help ML models do what? It is clear to me what you mean but please make it clear for the broad audience.

Literature Survey

- Line 56, 63, and 65: Why do you not use something like \citet to use the author’s name. Reading, for example, “was introduced by [11]” feels awkward.
- Line 90: You do not thoroughly introduce abbreviations before using them. Please always write a word out before using abbreviations. Latex, which you seem to be using, offers a range of solutions to automate this.
- You write some titles with capital letters like “Literature Survey” and some without like “Model evaluation and comparison”. Please use one style.

Background Information

- Line 105: Please provide evidence that syntactic information is also encoded in vector representations.
- Is Figure 1 really made by you? If not, you need to quote your source and check if you have the rights to use this figure.

Data pre-processing

- How did you recognize whether an Tweet was written in Arabic? Please include your answer into the manuscript.

Model evaluation and comparison

- Line 192: Redundant explanation of what the total number of words recognized is.
- Line 202, 203, 206, 212, 221: Grammatical errors.
- GitHub link is not visible and should not be placed in the footnote like this.
- Line 223: Why do you choose to evaluate a word not recognized by ArabWordVec? You answered that you want to show the poorer performance of recognizing words of the other models. However, you already made clear that Arab2Vec recognizes more words. This suffices. This section is about assessing word similarity. Please pick another word.
- Line 224: Why do you write Figure (4) and everywhere else you do not write the Figure number in parentheses?
- Line 226: Why is there so much white space?
- Figure 4: The emojis are close to not being readable. As per my first review, I encourage you to ditch the black background and choose a white background. Also, your figures look cramped, as if someone resized them without keeping the original aspect of the image. The letters look way too wide. So do the emojis. They should be round but appear oval. Please change this.

Experimental comparison of the models

- Line 274, 276, 277: Grammatical error. Also, you write Table with capital letters. Before you wrote it with small letters. Please stay consistent. I prefer it the capitalized version.
- Table 3. The English word “bus” is linked to the Arabic plural of bus. Please change the English word to busses or the Arabic word from albaaSaat to albaaS.
- Table 3: The word Arabic word for Muhammad looks like the Arabic letters “kmd”. Is it due to the font that the first letter looks like a “k” instead of a “muHa”?
- Line 285: Same as above, you inconsistently abbreviate or write out words.
- Line 286: Grammatical error.
- Figure 8. The word in the social media cluster that receives and extra explanation is impossible to read for me. It seems half-cut off.
- Line 287 and 288: Grammatical error.

Conclusion

- Line 301 and 312: Grammatical error.
- Table 5. Missing period after text. Missing explanation what NS, LR, SVC, and GB mean. See comments on abbreviations from above. You inconsistently use 1,2, or 3 digits after the period. Why do you write all performances of Arab2Vec Skip Gram in italics. With SVC, e.g., it is does not have the best performance.
- Table 6: See Table 5. The table needs to be understood without looking into the manuscript. This is not possible. What is ML what is DL? Be specific.
- Table 8 and 9 miss indications of the best performing Word2Vec models and in Table 8 you are missing a period in the table text.

Missed points from last review

Some points may repeat compared to points made above.

- Example for dialects still not in manuscript.
- Inconsistencies in lower- and upper cases in section titles persist.
- The authors answered how they tokenized their data but did not describe it in the manuscript. All technical details should be listed in the manuscript. Readers should not require to open a GitHub repository to understand what you did. The same applies to hyper parameters of the word2vec models and discussions on windows size and reasons for selecting tri-grams.
- You still use vertical lines in your tables which I discouraged you from using giving a reference.
- Even gray background is hard to read. In the reference I gave, it is clear that much brighter background is required for readability like white. Please follow the instruction in the reference or give a counter reference but do not ignore this point.
- Please include information on using k-means clustering in the manuscript.
- There is a misunderstanding on which highlighting method should be used in Tables to indicate the best performing model. Using bold is perfectly fine for me it should just be consistent. In this second version, Table 8 and 9 are missing such highlighting. Digits are still not rounded to two digits.
- There is a mess on whether you show result on the validation or test set. You still write validation set in Table 5, 8, and 9. Please fix this.
- Please describe your classification set up. How did you split your data. Was your dataset balanced? As mentioned above, it is not enough to describe these details only in code. Everything should be at one place.
