# Supplementary material for: Arab2Vec: An Arabic word embedding model for use in Twitter NLP applications
Source: PLoS One. 2025 Aug 29;20(8):e0328369. doi: 10.1371/journal.pone.0328369 (PMC12396693; doi:10.1371/journal.pone.0328369)
Supplement: Attachment — Submitted filename: Review Response v5.docx [file pone.0328369.s004.docx]

Dear Editor-in-chief (PLOS ONE):

I am pleased to submit our revised article titled “Arab2Vec: An Arabic word embedding model for use in Twitter NLP applications’’ by Abdelrahman Hamdy, Ayman Youssef and Conor Ryan for your consideration.

 Write the contributions in a standard manner. For instance, avoid starting with phrases like is an open-source model that was.... Instead, rephrase as Our model is an open-source.... Revise all contributions accordingly.

The section describing the work contributions is rewritten according to the reviewer comments. All contributions were rephrased.

“The proposed model has several advantages over other models in the literature. These can be summarized as follows: 32• It is an open-source model trained on 186M tweets and, therefore, can recognize

more words (2,027,042, approximately 33%) than previous models;

• It recognizes COVID-19-related words and emojis with higher accuracy as it was

trained on a newer data set; 36• It has a variant trained using negative sampling. This is an advantage for our work, as we will see from the results that this variant achieves higher accuracy than the previous model.

• It exhibits better performance than the two state-of-the-art models from the

literature (AraVec [9], AraWordVec [10] ) on the two tested data sets (COVID-19,

ASTD).”

- Refer to the section names in the last paragraph of the introduction for easy navigation. For example: The paper is organized as follows: Section 1 presents a literature survey, Section 2 describes the data collection process, and Section 3 discusses the data preprocessing conducted on the collected dataset. Ensure to use clickable references for section names.

The last paragraph of the introduction was modified depending on the reviewer comments

Section 1, which contains a literature survey, before moving to Section 2, which describes the data collection process, and Section 3, which discusses the pre-processing conducted on that set. Section 4 presents a qualitative comparison between the model and two other well-known models from the literature using publicly available data. Next, Section 5 presents a quantitative experimental comparison between the proposed model and models from the literature. Finally, Section 6 provides some conclusions and describes some future work.

- Improve the quality of the diagrams. Use high DPI versions, as the current ones appear blurry, particularly the text.

All images of the manuscript were revised. Some images just needed resizing to make them clearer, other images needed enhancement using online tools from the internet. We are not able to get image clearer from software. However, clear description of figures with tables for every word is added into manuscript for clarity in previous review rounds.

- Consider adding recent models in the discussion or future work sections. For example, mention that Large Language Models can be fine-tuned to produce embeddings for these tasks.

A new paragraph has been added to the discussion section concerning the capabilities of large language models.

“An alternate approach would be to employ a Large Language Model (LLM) to perform this task. In particular, the ability of LLMs to create context-aware representations is attractive. However, a dedicated word embedding model like the one proposed here can reasonably be expected to be more efficient and resource-friendly than fine-tuning a large language model for word embedding tasks. Future work will explore this trade-off further.”

- Overall, improve the English language throughout the manuscript. For instance, in the Abstract, revise sentences like The authors of this article provide Arab2Vec as an open-source project for users to employ in any research-related work. to We provide Arab2Vec as an open-source project for researchers to use in various applications.

The entire manuscript was revised to add clarity to the text. Several sentences were rewritten, other were modified for clarity.
