# Supplementary material for: Arab2Vec: An Arabic word embedding model for use in Twitter NLP applications
Source: PLoS One. 2025 Aug 29;20(8):e0328369. doi: 10.1371/journal.pone.0328369 (PMC12396693; doi:10.1371/journal.pone.0328369)
Supplement: Attachment — Submitted filename: Reviewer Response v6.docx [file pone.0328369.s005.docx]

Dear Editor-in-chief (PLOS ONE):

Please see the responses to the final comments from the reviewers.

*In particular, the quality of Figure 2 needs improvement, as the current version lacks the visual clarity expected for publication. The figure should be recreated to enhance its readability, method overall steps and presentation.*

Figure 2 was recreated to give more clarity to the steps involved in the work.

*Furthermore, although the introduction has been improved, the section discusses the paper’s contributions would benefit from further refinement. More clearly discussing the key findings, remove the references inside contributions and emphasizing the novelty of the work will help readers better understand the significance and impact of the research.*

This part has been completely rewritten, although we have kept the citations in place as this is where these data sets are mentioned.

*Finally, the manuscript should be carefully proofread to correct any remaining language issues and ensure consistency in style and formatting throughout.*

The manuscript has been proofread once more and checked with Grammarly, which returns a score of 99/100.
